# A Rare Case of a Neuroendocrine Tumor With Intracardiac Metastasis: Management and Literature Review

**DOI:** 10.7759/cureus.84538

**Published:** 2025-05-21

**Authors:** Brandon Wilkins, Pranali Pachika, Rohit Kumar

**Affiliations:** 1 Internal Medicine, University of Louisville, Louisville, USA; 2 Medical Oncology and Hematology, University of Louisville, Louisville, USA

**Keywords:** carcinoid syndrome, carcinoid tumor, intracardiac metastasis, neoplasm, neuroendocrine tumor

## Abstract

Neuroendocrine tumors (NETs) are rare neoplasms originating from the neuroendocrine system. While they can develop sporadically throughout the body, they are mostly found in the gastrointestinal tract, respiratory tract, pancreas, and thymus. NETs can be classified into low-grade indolent tumors, intermediate-grade tumors, or high-grade aggressive cancers. These tumors have the potential to metastasize to various parts of the body, with the liver being the primary site for metastases. However, it is extremely rare for NETs to metastasize to the heart. Here, we present a unique case of intracardiac metastasis from a pelvic NET in a 54-year-old male patient.

## Introduction

Neuroendocrine tumors (NETs), also called neuroendocrine neoplasms, are rare neoplasms that derive from the neuroendocrine system. The neuroendocrine system is composed of glands and nerves and is responsible for making hormones and releasing them into the bloodstream. NETs account for nearly 0.5% of all newly diagnosed cancers [1]. In the United States, the annual age-adjusted incidence of NETs is 6.98 per 100,000 persons which is an increase from 1.09 per 100,000 persons in 1973 [2]. It has been estimated that over 12,000 individuals in the United States are diagnosed with NETs annually, and approximately 175,000 people in the United States are living with NETs [3].

NETs can develop sporadically anywhere in the body, but they are mostly found in the gastrointestinal tract, respiratory tract, pancreas, and thymus [4]. Based on histology, clinical behavior, and proliferation rate, NETs are typically categorized similarly to lymphomas as either low-grade indolent tumors, intermediate-grade tumors, or high-grade aggressive cancers [1]. Patients with low-grade indolent tumors often present with nonspecific symptoms, leading to delays in diagnosis. Population-based studies report that up to 21% of patients are found to have metastases at the time of diagnosis due to these delays [5]. When metastasis occurs in NET patients, the liver is the primary site, often originating from the small intestine [6]. Cardiac metastasis (CM) of NETs is very rare, with an incidence estimated between 1% and 4%. However, this number is likely underestimated as many patients are asymptomatic and do not undergo routine cardiac imaging during tumor re-staging [7,8]. In 53% of the cases, myocardial metastatic disease from neuroendocrine disease involved the left ventricle of the heart [8]. Here, we present a rare presentation of an intracardiac metastasis from an intermediate-grade NET.

## Case presentation

We describe the case of a 54-year-old man with a history of degenerative joint disease who was initially evaluated by Urology for erectile dysfunction. During a renal ultrasound, an incidental soft tissue mass was found in the right pelvis extending towards the midline measuring 10.7 cm x 13.7 cm, exerting mass effect on the bladder. There were also additional masses in the liver suggestive of metastatic disease. A subsequent CT scan of the chest, abdomen, and pelvis revealed a large heterogeneous soft tissue mass in the pelvis with extensive retroperitoneal and bilateral pelvic adenopathy. This mass, located external to the rectum and sigmoid colon, displaced the sigmoid colon to the right. It also showed extensive abdominal and bilateral pelvic metastatic adenopathy, as well as pulmonary nodules and right hilar adenopathy. The initial differential diagnosis included a large retroperitoneal sarcoma or a large exophytic gastrointestinal/colonic malignancy.

**Figure 1:**
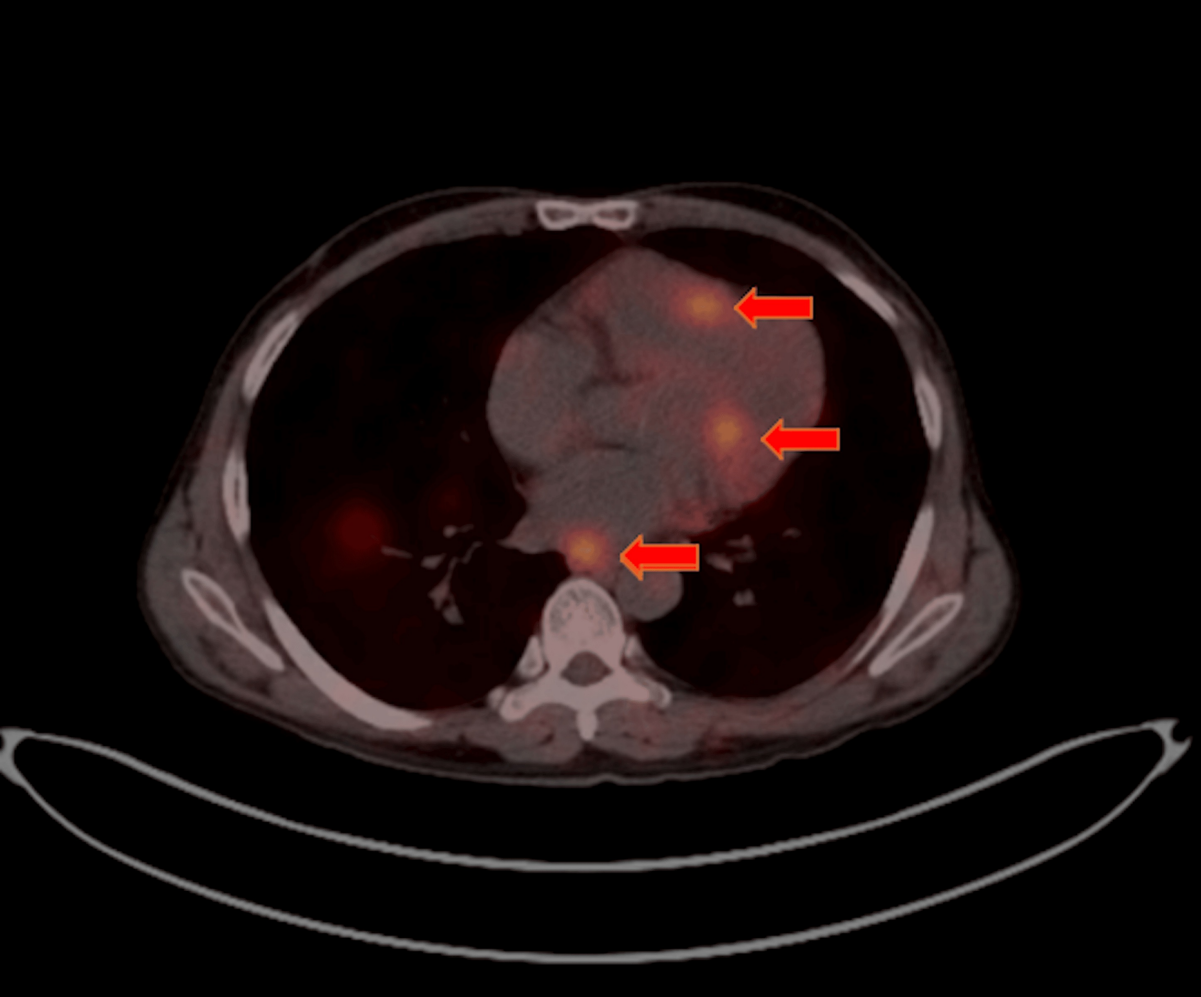
PET Dotatate showing the intracardiac metastasis from a pelvic neuroendocrine tumor

Interventional Radiology performed a biopsy of the pelvic mass, revealing a well-differentiated NET (carcinoma) with intermediate grade and a Ki67 index of 5-15%. A baseline Dotatate PET/CT scan confirmed findings of the CT scan showing a highly Dotatate-avid pelvic and abdominal mass and numerous moderately and highly FDG-avid metastases to the liver, pelvic, retroperitoneal, and mesenteric lymph nodes, left posterior apical pleural-based mass, and numerous Dotatate-avid skeletal metastases (see Figure 1). The baseline Chromogranin level was 281 (normal level is less than 311). The baseline 5-HIAA level was 1.7 (normal is less than 6.0).

**Figure 2:**
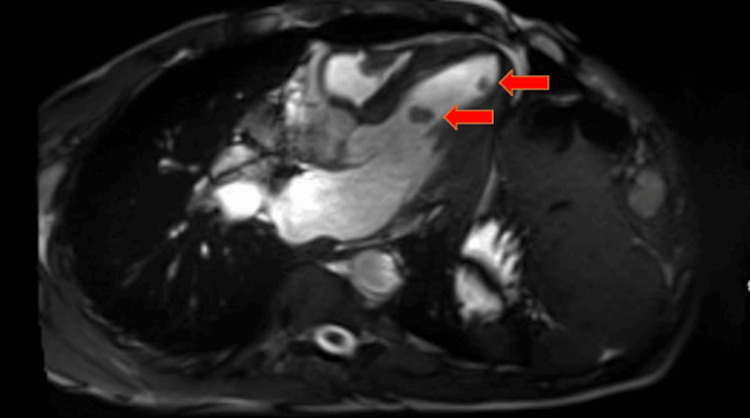
Cardiac MRI revealing two new hypodense filling defects within the left ventricular chamber concerning for intracardiac metastasis

**Figure 3:**
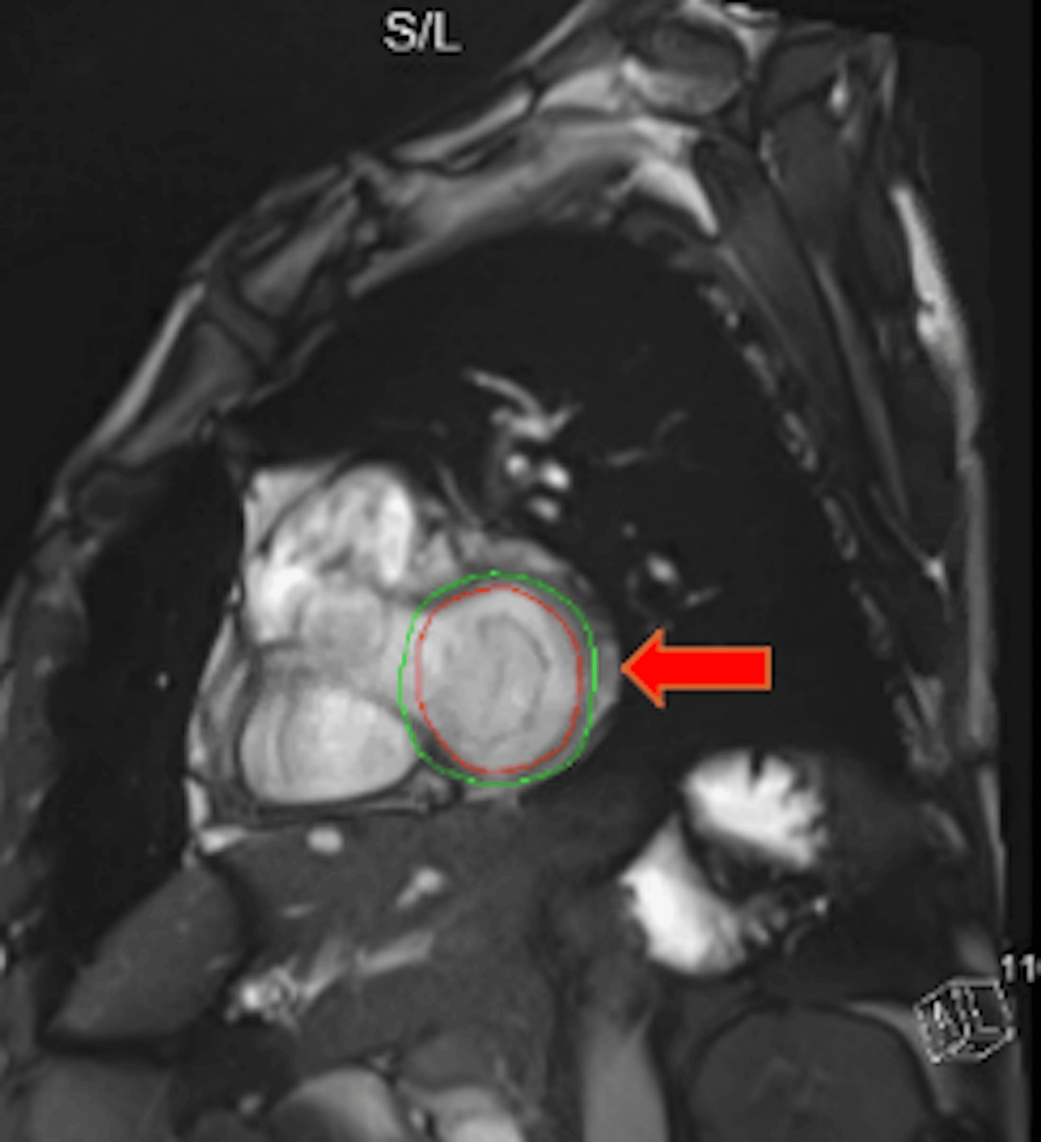
Cardiac MRI revealing intracardiac metastasis from a pelvic neuroendocrine tumor

Despite a high disease burden, the patient was able to carry out most daily activities without any issue. He had an ECOG of 1. The patient was subsequently started on lanreotide. After four months of lanreotide treatment, a follow-up CT scan of the abdomen and pelvis indicated disease progression. A repeat Dotatate PET/CT scan showed mild progression and concern for CM. A CT scan of the chest revealed two new hypodense filling defects within the left ventricular chamber, raising concern for intracardiac lesions. A transthoracic echocardiogram identified at least three mobile round echogenic masses attached to the endocardial surface of the left ventricle, measuring 1 to 1.4 cm in diameter. A cardiac MRI confirmed four intracardiac masses, with tissue characterization consistent with metastatic disease given the patient’s history of a NET (see Figures 2, 3). 

Given the rare site of metastasis, a multidisciplinary team, including cardiology, discussed the case. Although there is no data on the thrombogenic nature of intracardiac NETs, given the clinical risk the patient was started on Eliquis (apixaban) for stroke prevention. Cardiology and cardiac surgery evaluated the patient, and mass removal was deferred due to the metastatic nature of the disease and the possibility of regrowth. Systemic treatment was deemed the best approach, and the patient was started on Temodar (temozolomide) and Xeloda (capecitabine).

Three months after starting Temodar and Xeloda, a follow-up CT scan showed significant disease progression. Despite the intermediate grade histology from the biopsy, the clinical course suggested aggressive disease, prompting a switch in treatment to carboplatin and etoposide. After four cycles of carboplatin and etoposide, interim scans showed stable disease. However, the patient later experienced disease progression again and was started on peptide receptor radionuclide therapy (PRRT) using Lutetium and his disease remains stable on current management.

## Discussion

NETs rarely metastasize to the heart, with an incidence reported to be between 1% and 4% [7]. Due to its rarity, diagnosis can be challenging. For many patients, CM is diagnosed through imaging studies conducted to monitor changes in tumor burden. A large retrospective study conducted at the Royal Free Hospital in London, UK, reported that 40% of patients were asymptomatic, 48% experienced shortness of breath, and 12% had palpitations [7]. Patients with metastatic neuroendocrine disease to the heart may also exhibit symptoms typical of carcinoid syndrome, such as flushing, diarrhea, and dyspnea, due to elevated serotonin levels. In our patient, CM was discovered incidentally during interval imaging performed to assess disease status, and he remained asymptomatic from a cardiac standpoint.

When CM from a NET is suspected or identified, obtaining an echocardiogram is crucial. A two-dimensional echocardiogram can detect morphological changes to the tricuspid and pulmonary valves, while a Doppler echocardiogram and color flow imaging can provide a more precise evaluation of valvular dysfunction [9]. However, echocardiograms have limitations. While they are effective in visualizing larger CMs, they may overlook smaller ones [10]. Additionally, differentiating between tumors and thrombi can be challenging [10].

CT scans are another useful modality for evaluating CM, especially for investigating masses infiltrating the heart or pericardium [10]. MRI has historically been valuable for determining the anatomical localization of metastases but struggles to distinguish between benign and malignant masses [10]. Cardiac NET metastases typically express somatostatin receptors, making somatostatin receptor scintigraphy with OctreoScan a common choice for diagnosis and follow-up [10]. Recently, gallium-68 labeled somatostatin analogs (SSAs) for PET/CT, such as 68Ga-DOTA-TOC, 68Ga-DOTA-TATE, and 68Ga-DOTA-NOC, have become more popular due to their ability to detect smaller lesions [10]. In cases where noninvasive imaging modalities cannot establish a diagnosis, an endomyocardial biopsy may be considered to gather more information [11]. However, an endomyocardial biopsy should only be pursued if the results would impact the treatment plan and if the likelihood of obtaining a successful biopsy is high [11].

Due to the rarity of cardiac neuroendocrine metastasis, there is no standardized way of treating it. Strategies of treating cardiac neuroendocrine metastasis range from close follow-up to treatment with SSAs, surgery, and chemotherapy [10]. When cardiac neuroendocrine metastasis is identified, it is important to have a multidisciplinary approach from oncologists, surgeons, cardiologists, and radiologists. Many factors, such as the age of the patient, symptoms, histology, and grade of the disease, can have an impact on determining which treatment option is best for the patient. Of the options, there is no evidence that surgery for heart metastases provides a clear benefit for the patient or impacts their clinical outcome [10]. Chemotherapy is rarely an option in patients with low-grade indolent cardiac neuroendocrine metastasis but in high-grade aggressive cardiac neuroendocrine metastasis, it may offer some benefit [10]. 

So far, only one large retrospective study is available on cardiac neuroendocrine metastasis, with most of the reported literature consisting of case reports and case series. Liu et al. conducted a retrospective analysis showing the benefit of PRRT [7]. This study examined 3,500 patients with NETs treated at the Royal Free Hospital in the UK between January 1998 and January 2020. They identified 25 patients with CM. All CM identified developed from well-differentiated NETs. The median age of the CM cohort was 64 years, with the small intestine being the most common primary site (84%).

After CM diagnosis, nearly 90% (n = 22) of the patients received SSAs, and over 60% (n = 16) were treated with PRRT, either with 90Yttrium DOTATATE (n = 1) or 177Lutetium DOTATATE (n = 15). Two patients received molecular targeted therapy with everolimus (n = 1) or CC-223 (n = 1), and two patients underwent chemotherapy (5-FU/irinotecan; 5-FU/carboplatin/streptozocin). Among the patients, 48% had solitary cardiac lesions, while 52% had multifocal cardiac lesions, with the left ventricle being the most common site of metastasis (52%), followed by the right ventricle (44%). None of the patients underwent cardiac surgery, but one received external beam radiotherapy for inoperable CM for palliation. In the PRRT-treated NET CM cohort, the overall disease control rate was 75%, and a reduction in the size of CM was noted in 19% of these patients. Although the survival benefit of PRRT in the study did not achieve statistical significance (76.0 vs. 14.0 months, p = 0.196), likely due to the small number of patients, it showed promising efficacy in prolonging time to CM progression and life expectancy in this group. Grade 3 lymphopenia developed in 2 patients post-PRRT, but no Grade 4 hematological toxicities were identified. Additionally, no myelodysplasia or leukemia developed during long-term follow-up. Renal toxicities were recorded in two patients, while no hepatotoxicity was observed. All recorded adverse events were manageable and resolved with appropriate medical intervention. This analysis demonstrated that concomitant skeletal or pancreatic metastases and elevated N-terminal pro-B-type natriuretic peptide (NT pro-BNP) >2 × upper limit of normal were independent poor prognosticators [7].

This is the only study available so far demonstrating the benefit of PRRT for NET CM. The data on management modalities for NET CM remains limited, and more randomized clinical trials are needed to establish clear management guidelines. Due to the limited data, it is crucial to have multidisciplinary care team discussions at every step of management.

## Conclusions

Intracardiac metastases from a NET are very rare. Patients may experience cardiac symptoms such as shortness of breath, palpitations, and carcinoid symptoms or may remain asymptomatic. Our case illustrates a patient with an incidental finding of cardiac neuroendocrine metastasis, and we have detailed the management course for this patient. Given the rarity of this condition, there are no established guidelines for management, highlighting the need for further studies in this field.
